# Supplementary material for: Development and Validation of a Nomogram to Predict Overall Survival in Stage I–III Colorectal Cancer Patients after Radical Resection with Normal Preoperative Serum Carcinoembryonic Antigen
Source: Cancers (Basel). 2023 Nov 29;15(23):5643. doi: 10.3390/cancers15235643 (PMC10705739; doi:10.3390/cancers15235643)
Supplement: Supplementary file 1 [file cancers-15-05643-s001.zip › cancers-2611674-supplementary.pdf]

a

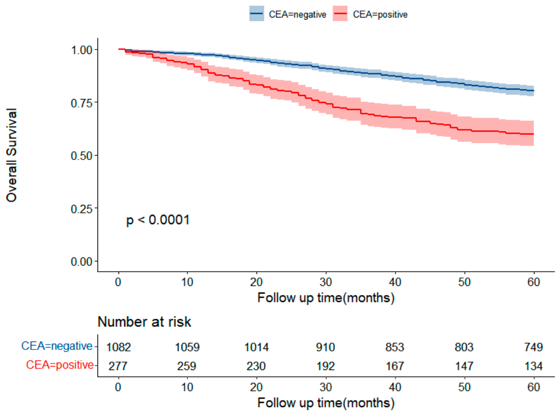
CEA=negative CEA=positive
Overall Survival
1.00
0.75
0.50
0.25
0.00
p < 0.0001
0 10 20 30 40 50 60
Follow up time(months)
Number at risk
CEA=negative 1082 1059 1014 910 853 803 749
CEA=positive 277 259 230 192 167 147 134
0 10 20 30 40 50 60
Follow up time(months)

b

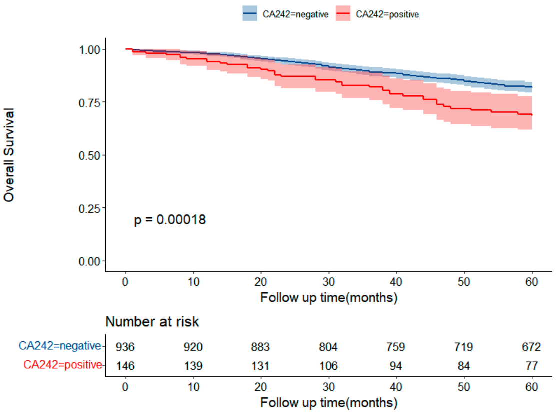
CA242=negative CA242=positive
Overall Survival
1.00
0.75
0.50
0.25
0.00
p = 0.00018
0 10 20 30 40 50 60
Follow up time(months)
Number at risk
CA242=negative 936 920 883 804 759 719 672
CA242=positive 146 139 131 106 94 84 77
0 10 20 30 40 50 60
Follow up time(months)

c

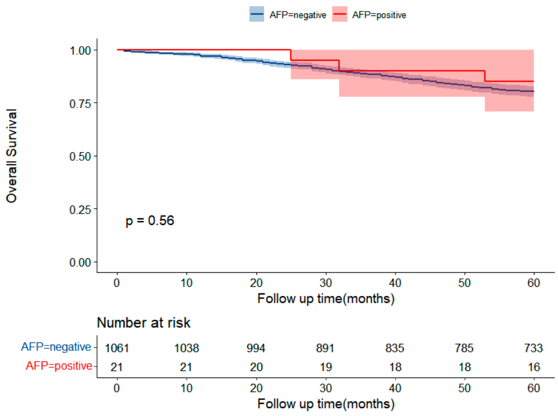
AFP=negative AFP=positive
Overall Survival
1.00
0.75
0.50
0.25
0.00
p = 0.56
0 10 20 30 40 50 60
Follow up time(months)
Number at risk
AFP=negative 1061 1038 994 891 835 785 733
AFP=positive 21 21 20 19 18 18 16
0 10 20 30 40 50 60
Follow up time(months)

d

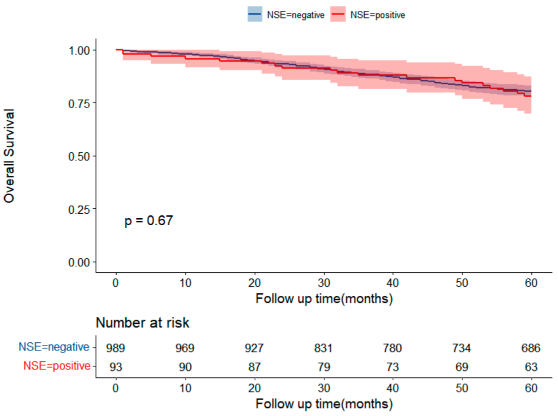
NSE=negative NSE=positive
Overall Survival
1.00
0.75
0.50
0.25
0.00
p = 0.67
0 10 20 30 40 50 60
Follow up time(months)
Number at risk
NSE=negative 989 969 927 831 780 734 686
NSE=positive 93 90 87 79 73 69 63
0 10 20 30 40 50 60
Follow up time(months)

e

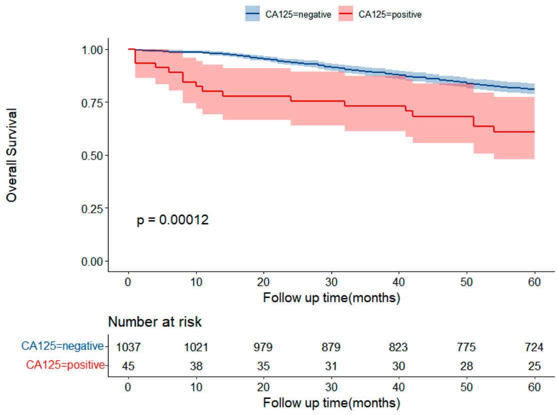
CA125=negative CA125=positive
Overall Survival
1.00
0.75
0.50
0.25
0.00
p = 0.00012
0 10 20 30 40 50 60
Follow up time(months)
Number at risk
CA125=negative 1037 1021 979 879 823 775 724
CA125=positive 45 38 35 31 30 28 25
0 10 20 30 40 50 60
Follow up time(months)

f

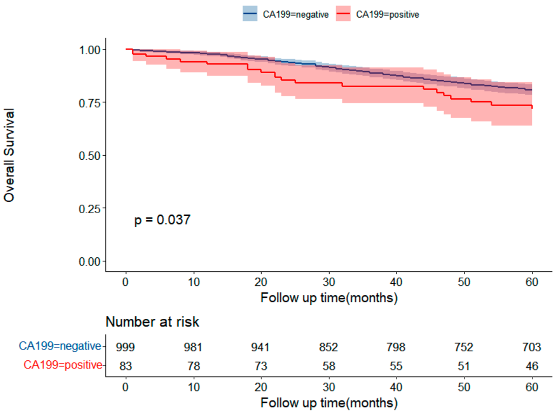
CA199=negative CA199=positive
Overall Survival
1.00
0.75
0.50
0.25
0.00
p = 0.037
0 10 20 30 40 50 60
Follow up time(months)
Number at risk
CA199=negative 999 981 941 852 798 752 703
CA199=positive 83 78 73 58 55 51 46
0 10 20 30 40 50 60
Follow up time(months)

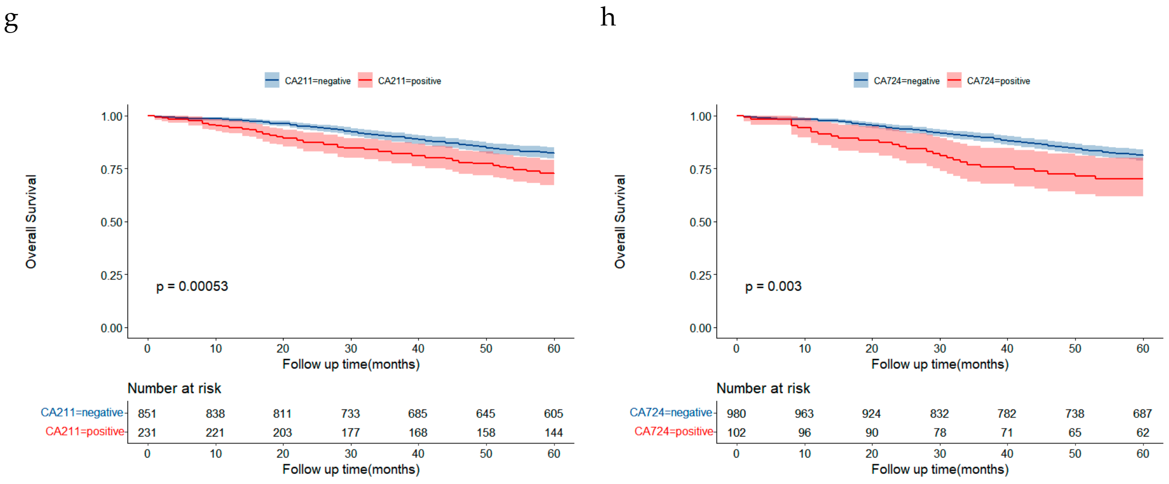

Supplemental Figure S1. Kaplan-Meier survival curves based on the level of preoperative tumor biomarkers.(A) Stratification of patients included in the study based on preoperative CEA levels.(B-H) Stratification of colorectal cancer patients undergoing radical surgery with normal preoperative CEA based on levels of other preoperative serum tumor markers.(CEA: carcinoembryonic antigen;CA242:carbohydrate antigen 242;AFP:alpha-fetoprotein;NSE:neuron-specific enolase;CA125:carbohydrate antigen 125;CA199:carbohydrate antigen 199;CA211:carbohydrate antigen 211;CA724:carbohydrate antigen 724).

Supplemental Table S1. The R packages used in this study.

Function	Packages
Random grouping（training set and validation set）	“caret” package
Predictors selection	“survival” and “MASS” packages
Nomogram and calibration curves	“rms” package
Receiver operating characteristic curves	“survivalROC” package
Decision curve analysis	“dcurves” package
Net reclassification improvement	“nricens” package
Integrated discrimination improvement	“survIDINRI” package
C-index and C-index change	“CsChange” package
Kaplan-Meier curves and Risk stratification	“survival”package

Supplemental Table S2. Baseline clinicopathologic characteristics.

Clinicopathological Features	cases	CEA（ng/ml）		p
	n（%）	≤10	＞10	
Sex				0.349
Male	798 (58.7)	628 (58.0)	170 (61.4)	
Female	561 (41.3)	454 (42.0)	107 (38.6)	
Age (median [IQR])	65 (58, 75)	65 (57, 75)	65 (59, 76)	0.109
Tumor Location				0.1
Right Colon	292 (21.5)	227 (21.0)	65 (23.5)	
Left Colon	425 (31.3)	328 (30.3)	97 (35.0)	
Rectum	642 (47.2)	527 (48.7)	115 (41.5)	
Histologic type				0.133
Grade I Adenocarcinoma	44 (3.2)	41 (3.8)	3 (1.1)	

Grade II Adenocarcinoma	966 (71.1)	769 (71.1)	197 (71.1)	
Grade III Adenocarcinoma	131 (9.6)	102 (9.4)	29 (10.5)	
Mucinous Adenocarcinoma	218 (16.0)	170 (15.7)	48 (17.3)	
pT stage				< 0.001
T1-2	318 (23.4)	298 (27.5)	20 (7.2)	
T3-4	1041 (76.6)	784 (72.5)	257 (92.8)	
pN stage				< 0.001
N0	778 (57.2)	654 (60.4)	124 (44.8)	
N1	355 (26.1)	267 (24.7)	88 (31.8)	
N2	226 (16.6)	161 (14.9)	65 (23.5)	
pTNM stage				< 0.001
I	264 (19.4)	248 (22.9)	16 (5.8)	
II	514 (37.8)	406 (37.5)	108 (39.0)	
III	581 (42.8)	428 (39.6)	153 (55.2)	
Perineural/Vascular invasion				0.005
No	1238 (91.1)	998 (92.2)	240 (86.6)	
Yes	121 (8.9)	84 (7.8)	37 (13.4)	
